# Mobile monitoring system detects the disease activity pattern and shows the association with clinical outcomes in patients with newly diagnosed Crohn’s disease

**DOI:** 10.1038/s41598-024-59914-7

**Published:** 2024-04-24

**Authors:** Yoo Jin Lee, Sang Gyu Kwak, Eun Soo Kim, Sung Kook Kim, Hyun Seok Lee, Yun Jin Chung, Byung Ik Jang, Kyeong Ok Kim, Jeongseok Kim, Hyeong Ho Jo, Eun Young Kim

**Affiliations:** 1 Department of Internal Medicine, Keimyung University School of Medicine, Daegu, South Korea; 2 Department of Medical Statistics, Catholic University of Daegu School of Medicine, Daegu, South Korea; 3 Department of Internal Medicine, School of Medicine, Kyungpook National University, 130 Dongdeuk-ro, Jung-gu, Daegu, South Korea; 4 Department of Internal Medicine, Yeungnam University College of Medicine, 317‑1 Daemyung 5 Dong, Daegu, 705-703 South Korea; 5 Department of Internal Medicine, Catholic University of Daegu School of Medicine, Daegu, South Korea

**Keywords:** Gastrointestinal diseases, Health care

## Abstract

We aimed to determine whether Crohn’s disease (CD) activity patterns assessed via a web-based symptom diary can help predict clinical outcomes in patients with newly diagnosed CD. Patients diagnosed with CD within the preceding 3 months were prospectively enrolled at four tertiary centers. All patients recorded their symptoms on a website using a smartphone at least once a week. The index outcomes were disease-related admission and surgery during follow-up. The disease activity from enrollment to outcome or last follow-up was reviewed for pattern analysis. Cox regression analysis was used to identify the predictors of disease outcomes. A total of 102 patients were enrolled. During a median follow-up period of 42 months, 25 (24.5%) and 6 (5.9%) patients required admission and surgery, respectively. Poor activity pattern was an independent predictor of disease-related hospitalization (adjusted hazard ratio [aHR], 3.96; 95% confidence interval [CI] 1.5–10.45; *p* = 0.005). A poor activity pattern (aHR, 19.48; 95% CI 1.86–203.95; *p* = 0.013) and female sex (aHR, 11.28; 95% CI 1.49–85.01; *p* = 0.018) were found to be independent predictors of bowel resection. CD disease activity patterns monitored through the mobile monitoring system may help predict clinical outcomes, such as disease-related hospitalization and surgery, in patients with newly diagnosed CD.

## Introduction

Crohn’s disease (CD) is a chronic inflammatory disease of the gastrointestinal tract characterized by a relapsing and remitting course^1^. Without adequate medical treatment, CD often results in irreversible bowel damage. Therefore, monitoring of disease activity and timely treatment is of utmost importance to prevent the complications of CD.

Traditionally, monitoring of patients with CD is based on clinical symptoms. Crohn’s disease activity index (CDAI) or Harvey–Bradshaw index (HBI) are commonly used tools for assessing the symptoms of CD^2,3^. Symptom resolution assessed using patient-reported outcomes (PRO) is one of the targets of inflammatory bowel disease (IBD) management in the treat-to-target strategy^4^. Such monitoring is typically performed intermittently (3–4 times a year) during outpatient follow-up. However, intermittent PRO monitoring may not be adequate for assessing the long-term progressive nature of CD as several clinical symptoms that occur at home, school, or workplace are liable to be missed^5^. Hence, seamless monitoring using remote technology may be an ideal approach for monitoring CD activity.

Self-monitoring via mobile technology has gained unprecedented traction amidst the boom in the development of mobile health applications and restricted health services during the coronavirus disease 2019 (COVID-19) pandemic^6,7^. For IBD, there are several commercially or noncommercially available apps, such as GI Monitor, GI Buddy, myIBD, HealthPROMISE, and UCLA eIBD^8–10^. These apps provide PRO symptom tracking, food diaries, medication alarms, and disease information. However, not all these apps have been validated, and the benefits of these apps in the management of IBD are not clear. Furthermore, no study has evaluated the utility of disease activity patterns detected by apps in predicting disease outcomes in patients with IBD.

We previously developed and validated a web-based CD symptom diary that can be used by patients via smartphones^11^. After defining the activity pattern as good (downward or stable activity) or poor (upward or fluctuating activity), we found that poor patterns were independently associated with poor clinical outcomes such as disease-related hospitalization and surgery^12^. However, the role of this pattern analysis in predicting disease outcomes could not be determined because the incidence of outcomes was included within the period of activity in the pattern analysis^12^. Furthermore, disease duration varied widely between patients, which may have affected disease outcomes. Therefore, in the present study, we aimed to determine whether activity patterns recorded via a web-based monitoring system can help predict disease outcomes in patients with newly diagnosed CD.

## Methods

### Patients and study design

This was a prospective observational study conducted at four tertiary referral centers. Consecutive adult patients who were diagnosed with CD within the preceding 3 months from December 2016 to September 2018 were enrolled. CD was diagnosed according to the guidelines based on clinical symptoms, laboratory results, endoscopic findings, and imaging studies^13^. The location of the disease and its behavior were classified using the Montreal Classification^14^. Disease activity at enrollment was categorized using the CDAI score. Patients with ileostomy, those unable to use smartphones, and those who used a diary for no more than one month were excluded. All patients recorded their symptoms in a web-based Crohn’s disease symptom diary (CDSD) using their smartphones at least once a week. Demographic and clinical data, including age, sex, disease location and behavior, current medications, baseline disease activity, and serum C-reactive protein (CRP) levels at enrollment were collected. This study was approved by the Institutional Review Boards of all four participating hospitals (Kyungpook National University Hospital; Daegu Catholic University Medical Center; Yeungnam University Hospital; and Keimyung University Dongsan Medical Center). This study was registered at www.clinicaltrials.gov (NCT02760836) and the study protocol complied with the principles enshrined in the Helsinki Declaration. Written informed consent was obtained from all patients prior to enrollment.

### CD symptom diary and pattern analysis

**Figure 1:**
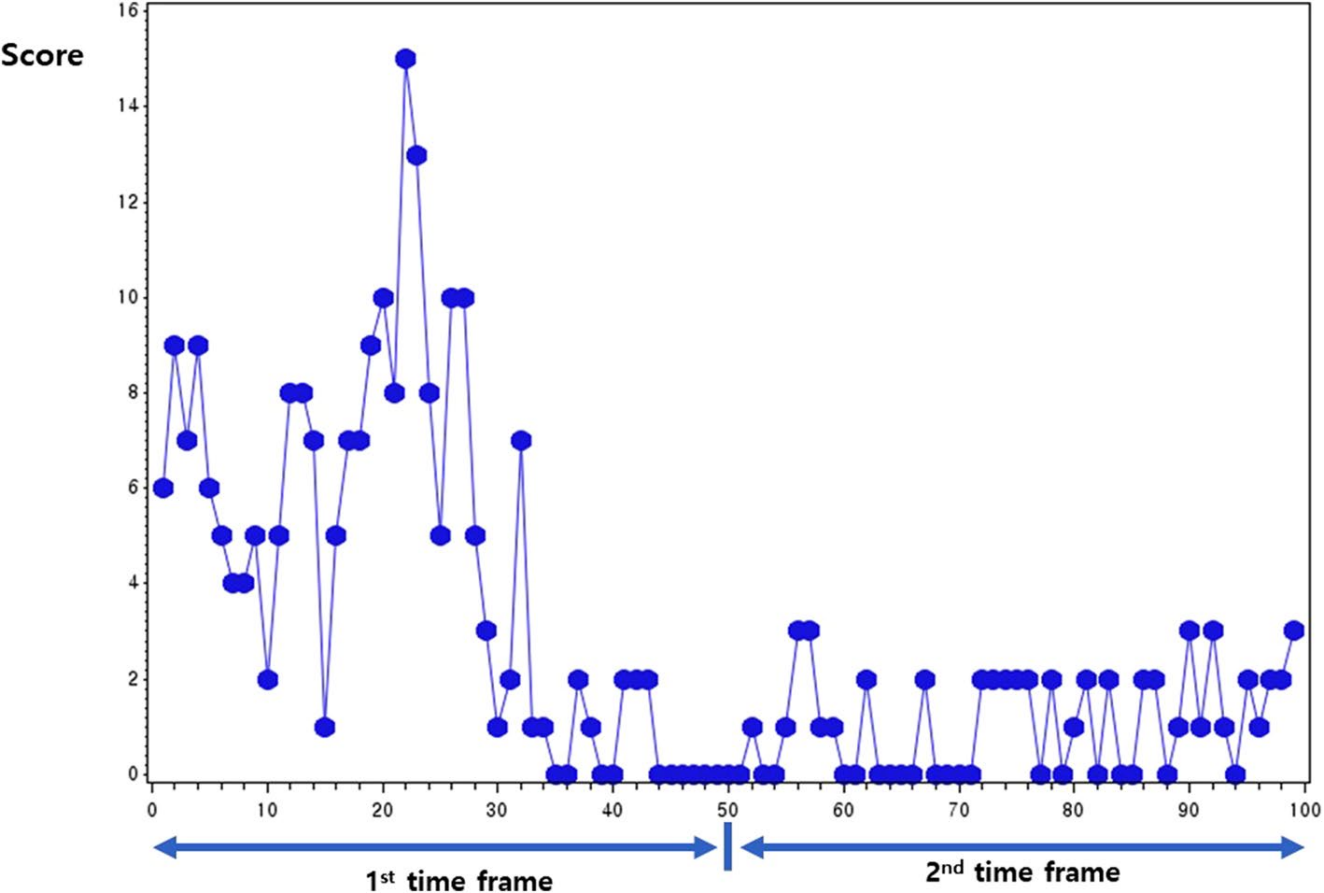
Graphical depiction of Crohn’s disease activity in the mobile monitoring system. The number on the x-axis indicates the number of activity records for the total follow-up period.

Variables in the CDSD were based on HBI, including the degree of abdominal pain, number of stools per day, general well-being, abdominal mass, and CD-related complications^11^. Patients recorded their symptoms in CDSD by clicking the relevant checkbox in the list of symptoms, and the score was automatically calculated^12^. In a previous study, the PRO score of CDSD showed a good correlation with the CDAI score^11^. Disease activity serially recorded via CDSD is graphically depicted in Fig. 1^12^. The X-axis indicated the number of whole activity records during study. This axis was divided into two frames by half of whole number of recordings; 1st frame and 2nd frame.

Pattern analysis has been described previously^12^. In brief, the activity pattern was classified as good or poor based on two factors: the degree of activity variation (maximum score–minimum score) and the tendency of activity (increasing, stationary, or decreasing). Based on these two factors in the two timeframes, there are 12 distinct patterns (Supplementary Table 1). When the variation is ≥ 6, it indicates a high variation, while a variation < 6 indicates a low variation. The follow-up period was divided into two timeframes, and variations were assessed within each timeframe. The tendency of activity was determined based on the difference between the mean scores of the two timeframes. Intuitively, increasing (higher mean score in the 2nd frame than that in the 1st) or persistently fluctuating activity (high variation of the scores in both time frames with a stationary trend [limited difference between the mean scores of each time frame]) were classified as poor patterns, whereas decreasing (higher mean score in the 1st frame than that in the 2nd) or stable activity (low variation of scores in both time frames with a stationary trend) were classified as good patterns. The details of the statistical calculations for the pattern analysis are provided in Supplementary Table 1.

The index clinical outcomes were disease-related hospitalization and surgery during the follow-up period. Anti-tumor necrosis factor (anti-TNF) use was assessed as an additional clinical outcome. For patients who experienced index outcomes during follow-up, disease activity from enrollment to the development of these outcomes was used. For patients who did not experience these outcomes, disease activity to the last follow-up was used for pattern analysis. Pattern analysis was separately performed for hospitalization and bowel resection because we surmised that activity patterns varied between these outcomes. The difference in the pattern for each outcome is explained in Supplementary Table 1. The final poor pattern group included patients showing poor patterns for hospitalization or bowel resection. In addition, as potential risk factors of hospitalization or surgery, CDAI at 3–6 months before the development of those outcomes were collected for patients who had index outcomes during follow-up. For patients without hospitalization or surgery, CDAI data at the last follow-up were collected.

### Statistical analysis

In our previous retrospective study of CDSD, 19.6% of poor pattern CD patients and 1.8% of good pattern CD patients underwent bowel resection surgery with a significant difference between these two groups^12^. Based on these figures and factoring in 80% power and a 2-sided α of 0.05, forty-eight patients were required in each group. Student’s *t*-test or Mann–Whitney U test was used for testing continuous variables, and Fisher’s exact test or χ^2^ test was used for categorical variables. Continuous variables are presented as mean ± SD or median (interquartile range). Kaplan–Meier analysis with the log-rank test was used to identify the predictors of the outcomes. Variables with a *p*-value ≤ 0.1 in the log-rank test were included in a multivariate analysis using the Cox regression hazard model. In addition, known risk factors of poor clinical outcomes^1^, such as disease behavior and age at diagnosis, were included as variables for multivariate analysis. CDAI scores during the follow-up period were also included for multivariate analysis; *p* values < 0.05 were considered indicative of statistical significance. All analyses were performed using R (v4.1.2; R Core Team 2021).

## Results

### Baseline characteristics of patients

Of the 135 patients who were newly diagnosed with CD, 102 (69 [67.6%] males) were included in the final analysis (mean age at diagnosis: 25.2 ± 9.6 years). Thirty-three patients were excluded due to the following reasons: seven patients did not record their symptoms; one patient refused to participate; 25 patients recorded data for less than 1 month. The baseline characteristics of the patients are summarized in Table 1. The most common location was L3 (ileocolonic, 44 [43.1%], followed by L1 (ileal, 32 [31.4%]). In terms of disease behavior, B1 (nonstricturing nonpenetrating, 77 [75.5%]) was more common than B2 (stricturing, 13 [12.7%] and B3 (penetrating, 12 [11.8%]). At enrollment, 60 patients were categorized as having clinically active disease (mild, 20 [19.6%]; moderate to severe, 40 [39.2%]), while 42 (41.2%) were considered in clinical remission.

**Table 1:** Baseline characteristics of the study population.

	Total N = 102	Poor pattern n = 43	Good pattern n = 59	*P*-value
Age at diagnosis, year, mean ± SD	25.2 ± 9.6	24.6 ± 8.5	25.7 ± 10.5	0.567
Male, n (%)	69 (67.6)	32 (74.4)	37 (62.7)	0.301
Education ≥ University, n (%)	66 (64.7)	25 (58.1)	41 (69.5)	0.33
Occupation, n (%)				0.298
Employed	24 (23.5)	12 (27.9)	12 (20.3)	
Unemployed	9 (8.8)	3 (7)	6 (10.2)	
Housewife	5 (4.9)	0	5 (8.5)	
Student	58 (56.9)	26 (60.5)	32 (54.2)	
Other	6 (5.9)	2 (4.7)	4 (6.8)	
Married, n (%)	23 (22.5)	9 (20.9)	14 (23.7)	0.925
Smoking, n (%)				0.989
Never smoker	81 (79.4)	34 (79.1)	47 (79.7)	
Exsmoker	12 (11.7)	5 (11.6)	7 (11.9)	
Current smoker	9 (8.8)	4 (9.3)	5 (8.5)	
Location, n (%)				0.658
L1	32 (31.4)	14 (32.6)	18 (30.5)	
L2	26 (25.5)	9 (20.9)	17 (28.8)	
L3	44 (43.1)	20 (46.5)	24 (40.7)	
UGI involvement, n (%)	5 (4.9)	3 (7)	2 (3.4)	0.716
Behavior, n (%)				0.492
B1	77 (75.5)	32 (74.4)	44 (74.6)	
B2	13 (12.7)	7 (16.3)	6 (10.2)	
B3	12 (11.8)	4 (9.3)	9 (15.3)	
Perianal disease, n (%)	54 (52.9)	24 (55.8)	30 (50.8)	0.768
Previous bowel surgery, n (%)*	12 (11.8)	4 (9.3)	8 (13.6)	0.728
Current medication, n (%)				
5-ASA	94 (92.2)	39 (90.7)	55 (93.2)	0.924
Corticosteroid	36 (35.3)	19 (44.2)	17 (28.8)	0.163
Thiopurine	77 (75.5)	34 (79.1)	43 (72.9)	0.628
CDAI at enrollment, mean ± SD	195.2 ± 101.8	223.9 ± 101	174.4 ± 98.1	0.015
Disease activity at enrollment, n (%)				0.016
Remission	42 (41.2)	11 (25.6)	31 (52.5)	
Mild	20 (19.6)	9 (20.9)	11 (18.6)	
Moderate to severe	40 (39.2)	23 (53.5)	17 (28.8)	
C-reactive protein, mg/dL, mean ± SD	2.9 ± 3.6	2.8 ± 3.6	2.9 ± 3.8	0.903
Follow-up period, months, median (interquartile range)	42 (18–51)	39 (16–51)	44 (20–51)	0.607

*These patients underwent surgery before or at the diagnosis of Crohn’s disease.

*CDAI* Crohn’s disease activity index, *SD* standard deviation, *ASA* aminosalicylate, *UGI* upper gastrointestinal.

**Figure 2:**
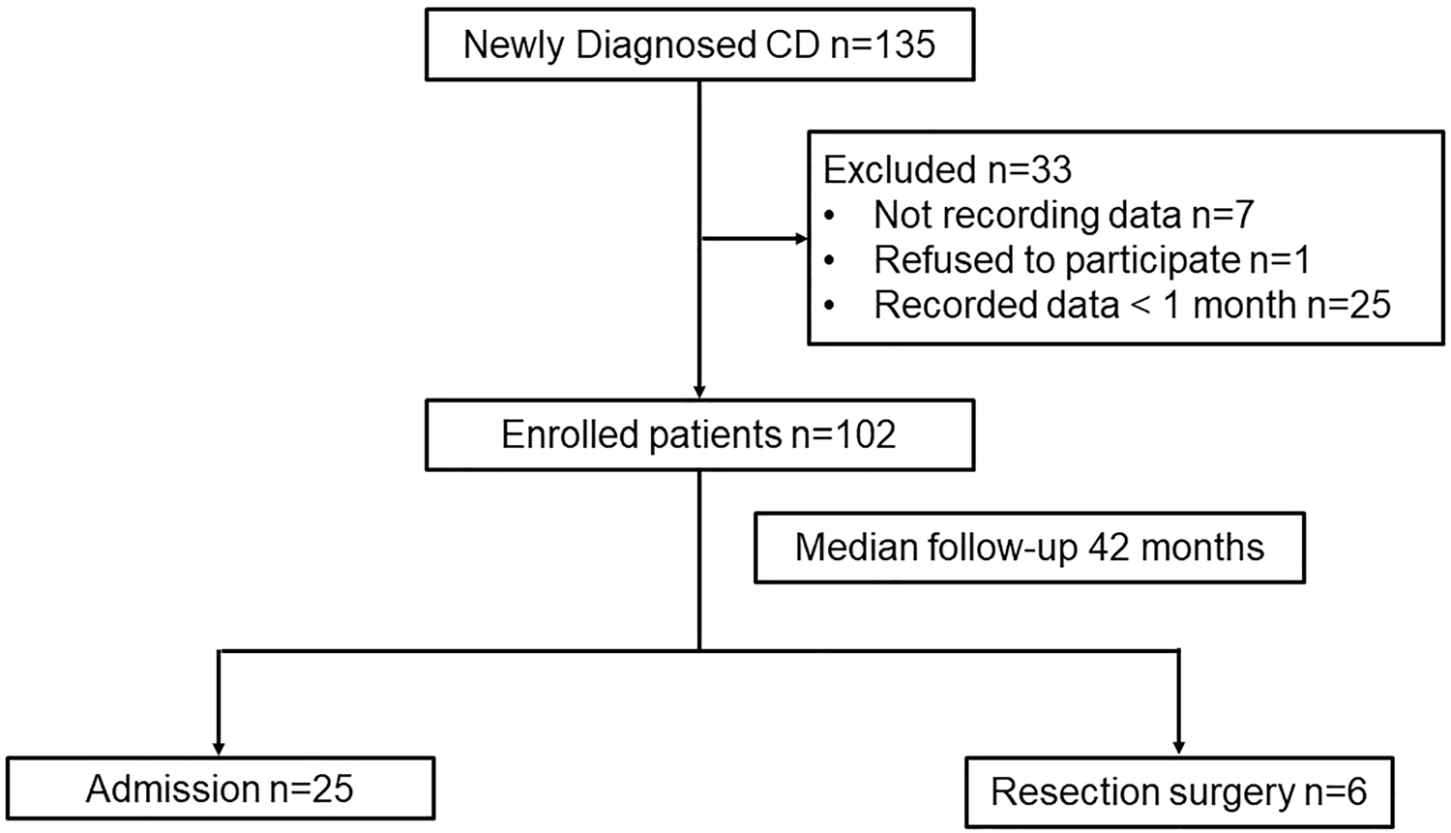
Flowchart depicting the inclusion of patients in the study.

During a median follow-up of 42 months (interquartile range, 18–51), 25 (24.5%) and 6 (5.9%) patients required disease-related hospitalization and surgery, respectively (Fig. 2). The median durations (month [interquartile range]) from the inclusion to hospitalization and to surgery were 6 (2–21) and 22 (17–27), respectively. We found no significant difference in the rate of hospitalization (28.9% vs. 24.5%, *p* = 0.665) and surgery (0 vs. 5.9%, *p* = 0.19) between 33 excluded-patients and 102 enrolled-patients. For hospitalization outcomes, pattern analysis revealed 36 patients with a poor pattern and 66 patients with a good pattern. Regarding bowel surgery outcomes, 37 and 65 patients were found to have poor and good patterns, respectively. Thirty patients showed a poor pattern with regard to both admission and surgery outcomes. Patients who had a poor pattern to admission or surgery were categorized as the final poor pattern group (43 [42.2%]), whereas the remaining patients showing a good pattern both to admission and surgery outcomes were categorized as the final good pattern group (59 [57.8%]). The baseline characteristics were not significantly different between the poor and good pattern groups, except for disease activity at enrollment. The mean CDAI score of the poor pattern group was significantly higher than that of the good pattern group (223.9 ± 101 vs. 174.4 ± 98.1, *p* = 0.015, Table 1).

### Predictors of clinical outcomes

**Figure 3:**
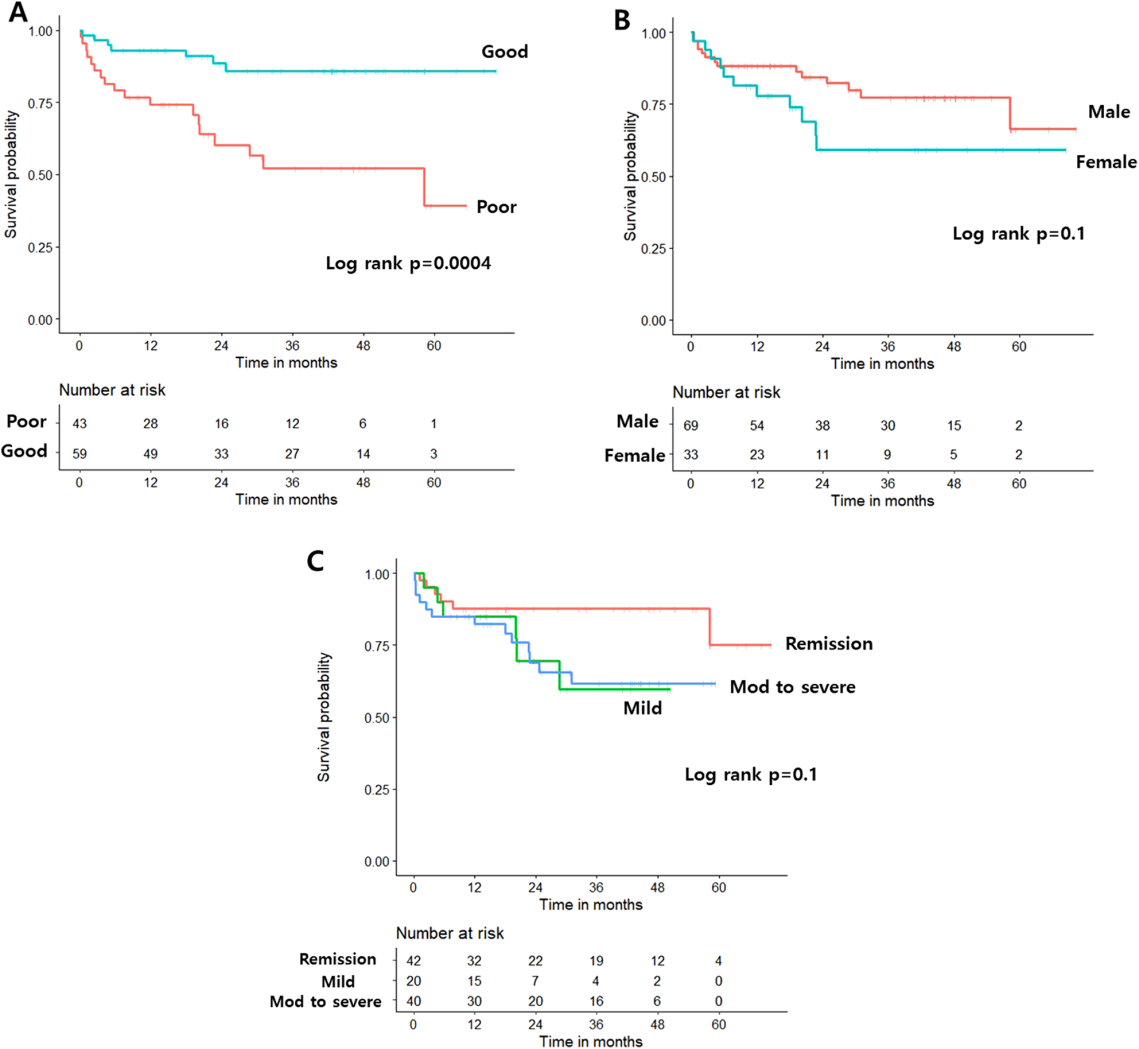
Kaplan–Meier analysis of the risk of disease-related admissions stratified by activity pattern (**A**), sex (**B**), and disease activity at enrollment (**C**).

**Figure 4:**
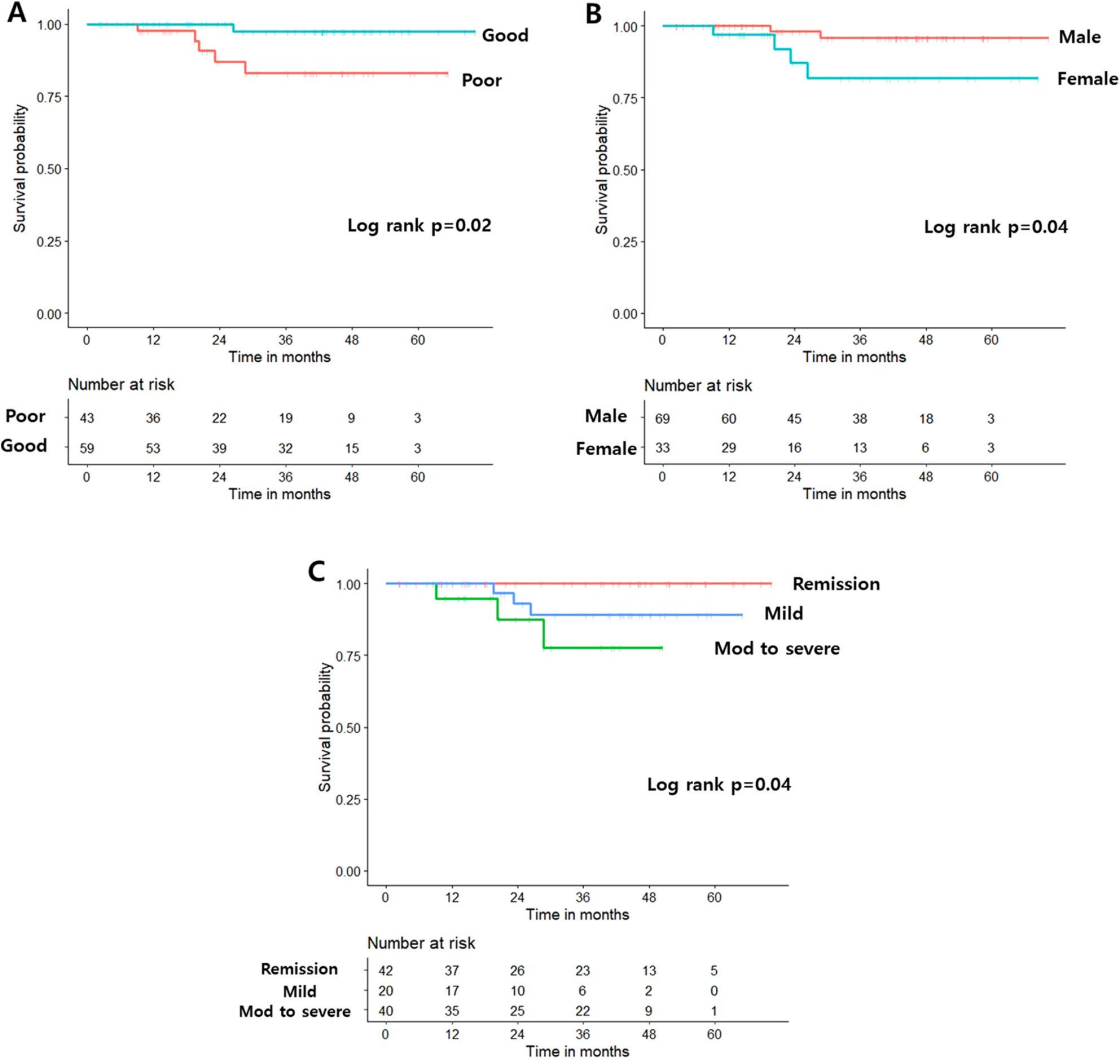
Kaplan–Meier analysis for the risk of bowel resection stratified by activity pattern (**A**), sex (**B**), and disease activity at enrollment (**C**).

Kaplan–Meier analysis revealed a higher risk of hospitalization in patients with a poor pattern than in those with a good pattern (log-rank *p* = 0.0004, Fig. 3A). Other baseline factors, including age at diagnosis, disease behavior and location, medication use, serum CRP level, perianal disease, previous surgery, and smoking, demonstrated no significant difference (see Supplementary Fig. 1A–H); however, sex (female vs. male, log-rank *p* = 0.1) and disease activity at enrollment (log-rank *p* = 0.1) demonstrated marginal significance (Fig. 3B–C). Kaplan–Meier analysis revealed that a poor activity pattern (log-rank *p* = 0.02), female sex (log-rank *p* = 0.04), and disease activity at enrollment (log-rank *p* = 0.04) were associated with a higher risk of surgery (Fig. 4A–C). The other baseline characteristics showed no significant difference (see Supplementary Fig. 2A–H).

We used a Cox proportional hazard regression model to control for confounding variables in the risk analysis for each outcome. A poor pattern was found to be an independent predictor of both disease-related hospitalization (adjusted hazard ratio, 3.96; 95% confidence interval [CI] 1.50–10.49, *p* = 0.005, Table 2) and bowel resection surgery (adjusted hazard ratio, 19.48; 95% CI 1.86–203.95, *p* = 0.013, Table 2). Female sex was an additional independent predictor of surgery (adjusted hazard ratio, 11.28; 95% CI 1.49–85.01, *p* = 0.018; Table 2). Regarding anti-TNF use outcome (23 [22.5%] patients), poor pattern showed a statistical significance in Kaplan–Meier analysis (Supplementary Fig. 3) while it failed to remain in Cox-regression analysis (Supplementary Table 2).

**Table 2:** Cox regression hazard analysis for the predictors of disease outcomes.

Outcomes	Variables	Univariate analysis		Multivariate analysis	
		Hazard ratio (95% CI)	*p*-value	Adjusted hazard ratio (95% CI)	*p*-value
Disease-related hospitalization	Poor pattern (vs. good)	4.25 (1.77–10.19)	0.0012	3.96 (1.50–10.49)	0.005
	Female (vs. male)	1.91 (0.86–4.21)	0.11	2.06 (0.84–5.08)	0.112
	Age at diagnosis < 17 years (vs. ≥ 17)	1.79 (0.71–4.51)	0.2	2.38 (0.84–6.71)	0.10
	CDAI at enrollment (CDAI < 150 vs. CDAI ≥ 150)	0.39 (0.15–0.99)	0.049	0.81 (0.29–2.24)	0.697
	CDAI during follow-up* (CDAI < 150 vs. CDAI ≥ 150)	0.42 (0.14–1.24)	0.115	0.52 (0.14–1.89)	0.323
	Behavior B1 (vs. B2 or B3)	0.89 (0.37–2.14)	0.79	0.90 (0.29–2.72)	0.856
Bowel resection surgery	Poor pattern (vs. good)	7.92 (0.92–67.84)	0.059	19.48 (1.86–203.95)	0.013
	Female (vs. male)	4.98 (0.91–27.24)	0.064	11.28 (1.49–85.01)	0.018
	Age at diagnosis < 17 years (vs. ≥ 17)	2.15 (0.39–11.78)	0.376	3.07 (0.45–20.66)	0.248
	CDAI at enrollment (CDAI < 150 vs. CDAI ≥ 150)	0.66 (0.14–3.32)	0.623	0.57 (0.09–3.46)	0.548
	Behavior B1 (vs. B2 or B3)	0.66 (0.12–3.61)	0.632	0.75 (0.11–5.12)	0.778

*CDAI at 3–6 months before outcome occurrence was collected for patients having surgery or admission during follow-up, while CDAI at last follow-up was collected for patients without events. This variable could not be analyzed for bowel resection surgery due to a small number of events. CDAI, Crohn’s disease activity index.

## Discussion

In this prospective multicenter study, the activity pattern described using a mobile monitoring system based on PRO was found to predict adverse outcomes in patients with newly diagnosed CD. A poor pattern was an independent predictor of disease-related admission and bowel resection. To the best of our knowledge, this is the first study to investigate the role of activity patterns in predicting disease outcomes in patients with CD.

Telemedicine or digital health technologies in the form of telemonitoring, tele-education, or telecounseling have not been found to be effective in improving disease outcomes in IBD, such as the risk of hospitalization or surgery and decreasing disease activity. In two randomized controlled trials, telemedicine was found to significantly reduce the hospitalization rate compared with standard care (traditional face-to-face encounters in the clinic) in IBD^15,16^; however, most other studies have found no difference in the hospitalization risk between telemedicine and standard care^17–21^. Additionally, no studies have reported the effects of telemedicine in decreasing the risk of surgery^15,16,18,22^. In a systematic review of randomized controlled studies investigating the effects of telemedicine on various outcomes compared with those of standard care, digital health intervention was found to be effective in reducing health utilization/cost; however, it had no effect on decreasing the relapse rate and improving treatment adherence^23^. This lack of effectiveness of digital health interventions in improving disease outcomes in IBD may be attributable to the relatively short follow-up for disease outcomes, mild disease activity in the study population, and nonspecific symptom-driven monitoring without objective measures (such as endoscopy or biochemical results) which are associated with better clinical outcomes^23^. Our findings suggest that the activity pattern based on a continuous assessment of PRO may be more useful than an occasional check-up of IBD symptoms in an outpatient clinic. In the present study, CDAI scores at enrollment (*p* = 0.697 for admission, *p* = 0.548 for surgery) or during follow-up (*p* = 0.323 for admission) were not found to predict disease outcomes (Table 2), supporting the potential advantage of continuous PRO monitoring over random assessment of CDAI. It is reasonable to surmise that patients with a poor activity pattern might benefit from early medical interventions, such as step-up therapy, based on an alarming notification in the remote monitoring telemedicine system. Further studies are required to determine whether digital health technologies using pattern analysis can help alter the disease course in patients with CD.

We analyzed data for anti-TNF use as another disease outcome. Although the poor pattern group showed a significantly higher risk of anti-TNF use than the good pattern group in the univariate analysis (Cox regression analysis *p* = 0.031, Supplementary Table 2; log-rank *p* = 0.03, Supplementary Fig. 3), the between-group difference was not statistically significant in the multivariate analysis after controlling for confounding factors (*p* = 0.124, Supplementary Table 2). Instead, CDAI at enrollment (*p* = 0.004) and disease behavior (*p* = 0.013) were found to be independent predictors of anti-TNF use (Supplementary Table 2). Although the reason for this disparate ability of pattern analysis for different clinical outcomes is not clear, the timing of the occurrence of the event is one of the plausible explanations. Anti-TNF initiation was observed earlier than the occurrence of admission or surgery (Supplementary Fig. 4); anti-TNF therapy was initiated in less than 1 year, whereas admission and surgery were generally observed in 20 months. The predictive ability of activity patterns for clinical outcomes in patients with IBD may manifest after a certain period. This suggests that pattern analysis might be useful for predicting long-term clinical outcomes. This finding needs to be confirmed in another independent cohort.

One of the key challenges in implementing telemedicine for IBD is the establishment of robust patient engagement^24^. In previous studies, approximately 50–70% of eligible patients refused or did not reply to the invitation, and the attrition rate was high (up to 32%)^15,17,20,21,25^. Most patients in previous studies had a longstanding disease with inactive or mild disease^15,17,18,21^. Patients in good condition are less likely to record their symptoms using an app because it may constantly remind them of their underlying disease^24^. The relatively high participation rate in our study (102/135, 75.5%) might be explained by a short disease duration (enrollment within 3 months after diagnosis) and the high proportion of patients with active disease; newly diagnosed patients or those with active symptoms are more likely to be interested in monitoring their disease activity.

The risk factors for surgery in CD include small bowel involvement, penetrating disease behavior, and smoking^26^. In an Asian cohort study, penetrating disease behavior was found to be an independent predictor of surgery in CD^27^. However, these variables were not identified as risk factors for surgery in the present study. This may be attributable to differences with respect to the characteristics of the study population and study design. For example, we excluded patients who could not use smartphones or those who recorded symptoms for less than a month. In addition, the small number of events (6 cases of surgery) might have reduced the statistical power. There are conflicting results regarding the influence of sex on outcomes. Some studies^28,29^ have found female sex as an independent risk factor for surgery in CD, which is consistent with our results.

One might argue that the CDSD use might be different between patients according to their clinical symptoms or well-being. For instance, sick patients might have reported their symptoms more frequently using CDSD than those who were doing well. To address this issue, we compared the median number of reporting symptoms between patients with surgery or hospitalization (25 [24.5%]) and those without these outcomes (77 [75.5%]). We found no significant difference in the median use number of CDSD between these two groups (47 [IQR 25–152] vs. 52 [25–141], p = 0.646) suggesting that patients’ clinical outcomes might not have significantly affected pattern analysis using CDSD.

The strengths of the present study include its prospective design, a homogeneous group of patients with newly diagnosed CD, and a relatively long follow-up period. Nonetheless, some limitations of this study should be acknowledged. The number of patients was relatively small. Although this study was conducted prospectively, the pattern analysis was performed retrospectively, which may have introduced an element of bias. In addition, the predictive ability of this pattern analysis needs to be validated in an independent cohort. As the patients were recruited from tertiary referral centers, the results might not be generalizable to all patients with CD. Lastly, although this study showed the potential usefulness of symptom-based assessment in predicting CD outcomes, there is a significant disconnect between patients’ subjective symptoms and objective measures such as biomarkers (CRP or fecal calprotectin) and endoscopic activity. Indeed, a study investigating the role of CRP in predicting patient prognosis showed a significant association between elevated CRP levels, even in patients with clinical remission, and subsequent CD-related hospitalization and intestinal resection during follow-up, suggesting the potential risk of a symptom-based approach in CD^30^. In addition, there is an overlap of symptoms between CD and other types of irritable bowel syndrome. Therefore, the results of this study need to be interpreted with caution.

In conclusion, the activity pattern based on a mobile monitoring system may help predict clinical outcomes, such as disease-related hospitalization and bowel resection surgery, in patients with newly diagnosed CD, thus underlining the potential role of telemedicine activity patterns in the management of IBD. Such remote monitoring with unique pattern analysis could be clinically relevant, particularly in the era of the COVID-19 pandemic. Further studies are required to ascertain whether management based on such pattern analysis can alter the disease course in patients with CD.

### Supplementary Information

Supplementary Information.

## Data Availability

The datasets used and analyzed during the current study are available from the corresponding author on reasonable request.

## References

[1] Torres J, Mehandru S, Colombel JF, Peyrin-Biroulet L (2017). Crohn’s disease. Lancet.

[2] Sandborn WJ (2002). A review of activity indices and efficacy endpoints for clinical trials of medical therapy in adults with Crohn’s disease. Gastroenterology.

[3] Harvey RF, Bradshaw JM (1980). A simple index of Crohn's-disease activity. Lancet.

[4] Turner D (2021). STRIDE-II: An update on the selecting therapeutic targets in inflammatory bowel disease (STRIDE) initiative of the international organization for the study of IBD (IOIBD): Determining therapeutic goals for Treat-to-Target strategies in IBD. Gastroenterology.

[5] Pariente B (2011). Development of the Crohn’s disease digestive damage score, the Lemann score. Inflamm. Bowel Dis..

[6] Kim KO, Jang BI (2022). Management of inflammatory bowel disease in the COVID-19 era. Intest. Res..

[7] Park YE (2022). Korean association for the study of intestinal diseases guidance for clinical practice of adult inflammatory bowel disease during the coronavirus disease 2019 pandemic: Expert consensus statements. Intest. Res..

[8] Con D, De Cruz P (2016). Mobile phone apps for inflammatory bowel disease self-management: A systematic assessment of content and tools. JMIR MHealth UHealth.

[9] Chang S, Hamilton M, Lees C, Atreja A (2020). Mobile health in IBD: Enhancing care, one phone at a time. Inflamm. Bowel Dis..

[10] Van Deen WK (2016). Development and validation of an inflammatory bowel diseases monitoring index for use with mobile health technologies. Clin. Gastroenterol. Hepatol..

[11] Kim ES (2017). Development of a web-based, self-reporting symptom diary for Crohn’s disease, and its correlation with the Crohn’s disease activity index. J. Crohns Colitis.

[12] Kim ES (2018). Disease activity patterns recorded using a mobile monitoring system are associated with clinical outcomes of patients with Crohn’s disease. Dig. Dis. Sci..

[13] Park JJ (2017). Second Korean guidelines for the management of Crohn’s disease. Intest. Res..

[14] Satsangi J, Silverberg MS, Vermeire S, Colombel JF (2006). The Montreal classification of inflammatory bowel disease: Controversies, consensus, and implications. Gut.

[15] de Jong MJ (2017). Telemedicine for management of inflammatory bowel disease (myIBDcoach): A pragmatic, multicentre, randomised controlled trial. Lancet.

[16] Cross RK (2019). A randomized controlled trial of TELEmedicine for patients with inflammatory bowel disease (TELE-IBD). Am. J. Gastroenterol..

[17] Elkjaer M (2010). E-health empowers patients with ulcerative colitis: A randomised controlled trial of the web-guided ‘constant-care’ approach. Gut.

[18] Del Hoyo J (2018). A web-based telemanagement system for improving disease activity and quality of life in patients with complex inflammatory bowel disease: Pilot randomized controlled trial. J. Med. Internet Res..

[19] McCombie A (2020). A noninferiority randomized clinical trial of the use of the smartphone-based health applications IBDsmart and IBDoc in the care of inflammatory bowel disease patients. Inflamm. Bowel Dis..

[20] Akobeng AK (2015). Telephone consultation as a substitute for routine out-patient face-to-face consultation for children with inflammatory bowel disease: Randomised controlled trial and economic evaluation. EBiomedicine.

[21] Carlsen K (2017). Self-managed ehealth disease monitoring in children and adolescents with inflammatory bowel disease: A randomized controlled trial. Inflamm Bowel Dis.

[22] Miloh T (2017). Text messaging effect on adherence in children with inflammatory bowel disease. J Pediatr Gastroenterol Nutr.

[23] Nguyen NH (2022). Digital health technologies for remote monitoring and management of inflammatory bowel disease: a systematic review. Am J Gastroenterol.

[24] Plevris N, Lees CW (2022). Disease monitoring in inflammatory bowel disease: Evolving principles and possibilities. Gastroenterology.

[25] Cross RK, Cheevers N, Rustgi A, Langenberg P, Finkelstein J (2012). Randomized, controlled trial of home telemanagement in patients with ulcerative colitis (UC HAT). Inflamm Bowel Dis.

[26] Peyrin-Biroulet L (2012). Surgery in a population-based cohort of Crohn’s disease from Olmsted County, Minnesota (1970–2004). Am J Gastroenterol.

[27] Ng SC (2016). Early course of inflammatory bowel disease in a population-based inception cohort study from 8 countries in Asia and Australia. Gastroenterology.

[28] Munkholm P, Langholz E, Davidsen M, Binder V (1993). Intestinal cancer risk and mortality in patients with Crohn’s disease. Gastroenterology.

[29] Gupta N (2006). Risk factors for initial surgery in pediatric patients with Crohn’s disease. Gastroenterology.

[30] Oh K (2017). Elevated C-reactive protein level during clinical remission can predict poor outcomes in patients with Crohn’s disease. PLOS ONE.

