# Supplementary material for: Mobile monitoring system detects the disease activity pattern and shows the association with clinical outcomes in patients with newly diagnosed Crohn’s disease
Source: Sci Rep. 2024 Apr 24;14:9405. doi: 10.1038/s41598-024-59914-7 (PMC11043071; doi:10.1038/s41598-024-59914-7)
Supplement: Supplementary file 1 — Supplementary Information. [file 41598_2024_59914_MOESM1_ESM.pdf]

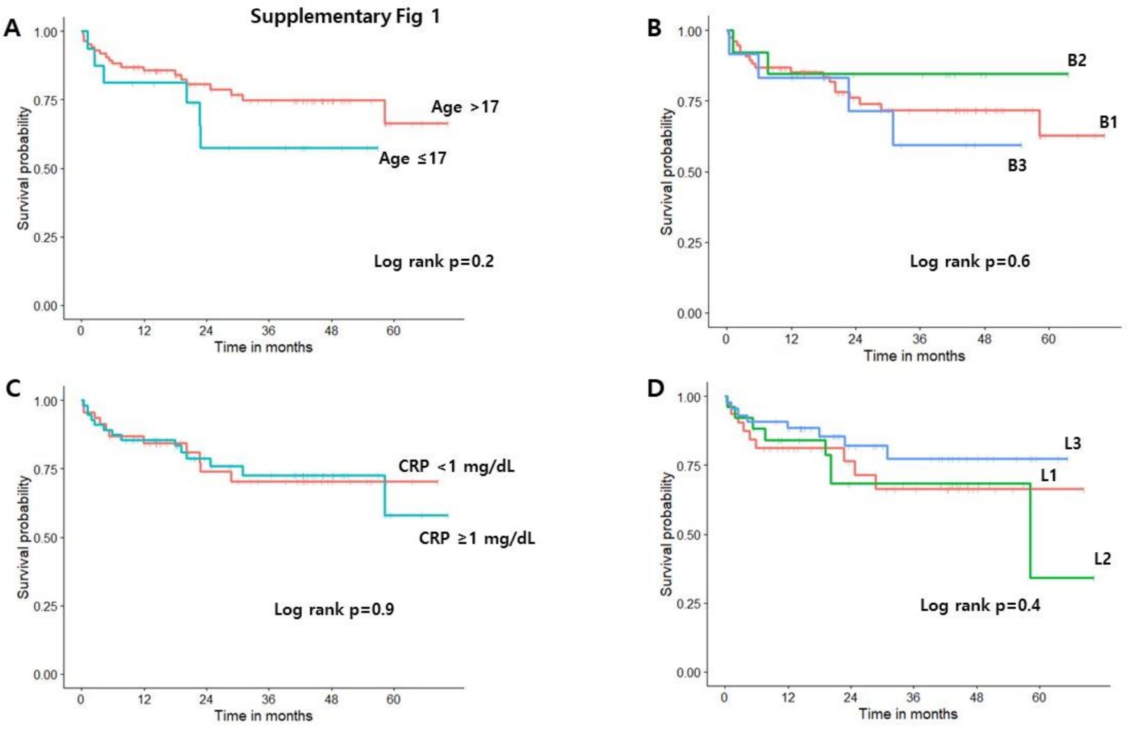

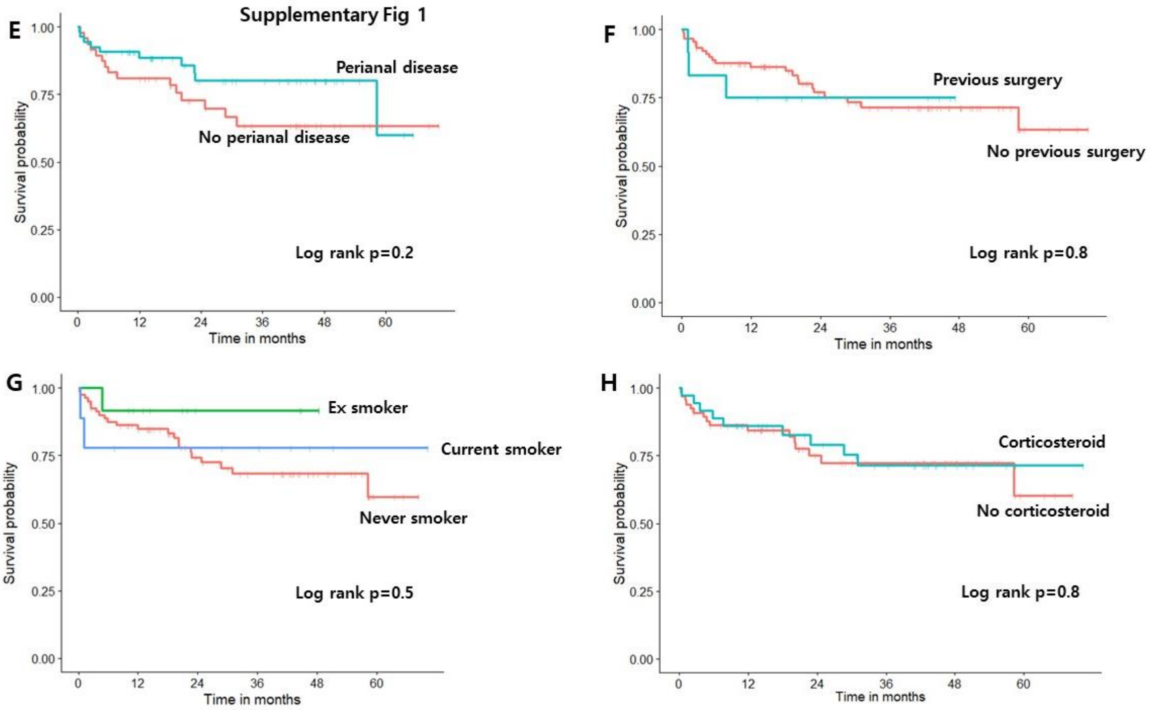

Supplementary figure 1. Kaplan–Meier analysis for the risk of disease-related admissions stratified by age (A), behavior (B), serum C-reactive protein level at enrollment (C), anatomical site (D), perianal disease (E), previous surgery (F), smoking (G), and corticosteroid use (H).

Supplementary Fig 2

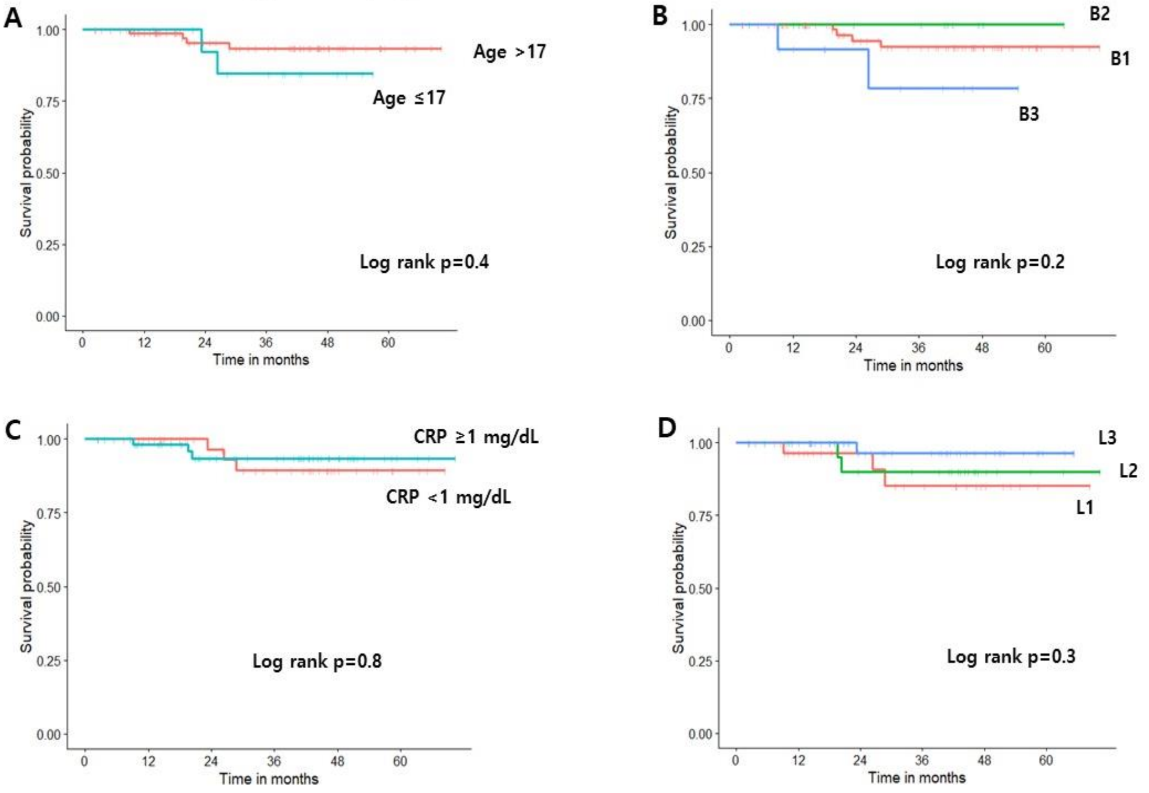

Supplementary Fig 2

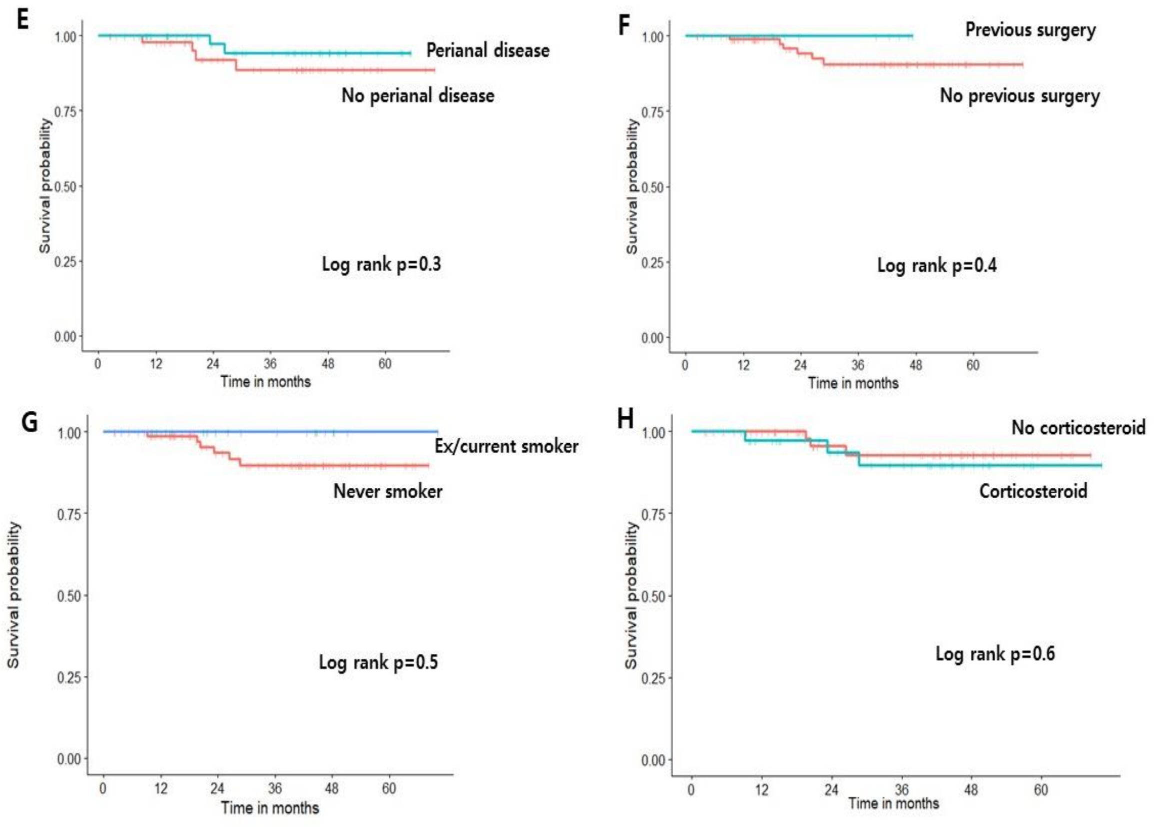

Supplementary figure 2. Kaplan–Meier analysis for the risk of bowel resection stratified by age (A), behavior (B), serum C-reactive protein level at enrollment (C), anatomical site (D), perianal disease (E), previous surgery (F), smoking (G), and corticosteroid use (H).

Supplementary Fig 3

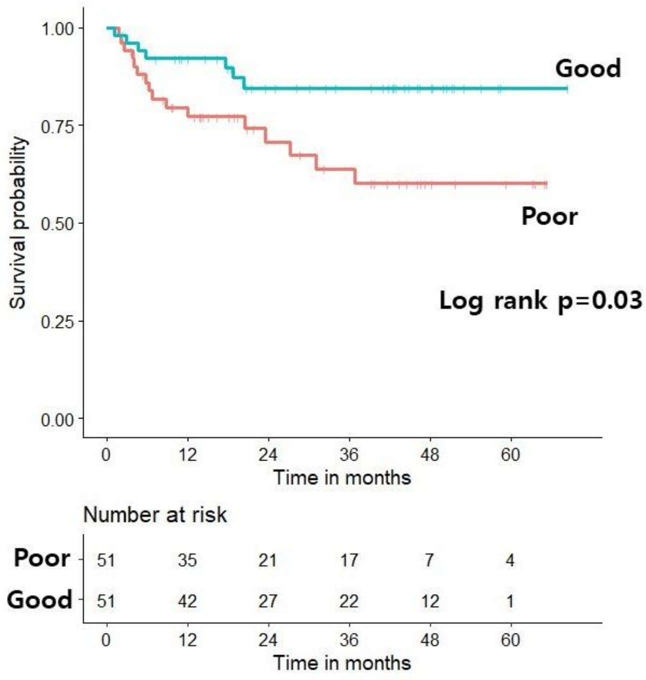

Supplementary figure 3. Kaplan–Meier analysis of the risk of anti-TNF use stratified by activity pattern.

Supplementary Fig 4

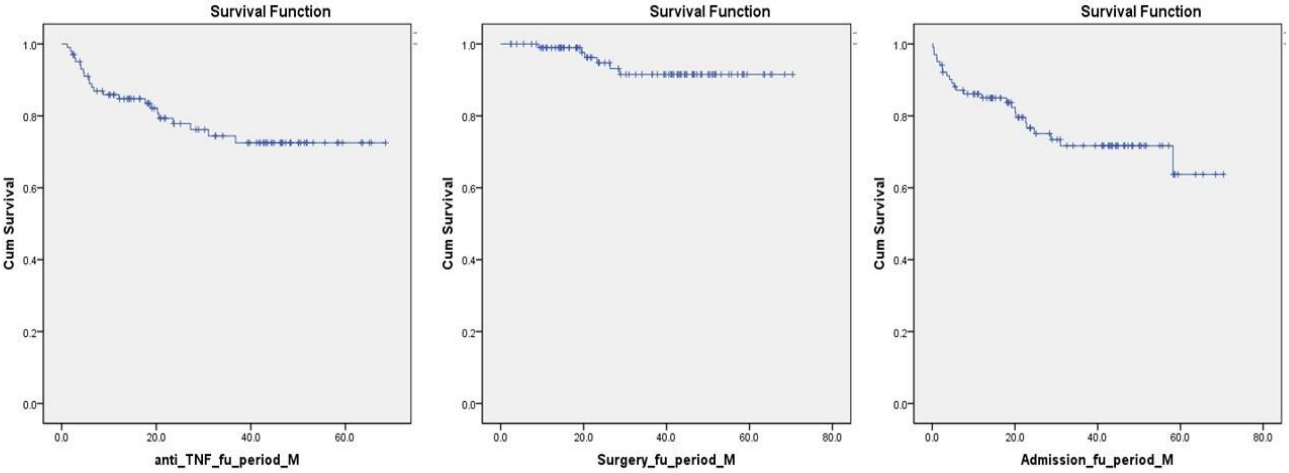

Supplementary figure 4. Cumulative risk of disease outcomes, including anti-TNF use, surgery, and admission during follow-up.

Supplementary Table 1. Graph pattern classification

Classification		diff < -a	-a ≤ diff < a	a ≤ diff
v1 < 6	v2 < 6	I	II	III
	v2 ≥ 6	IV	V	VI
v1 ≥ 6	v2 < 6	VII	VIII	IX
	v2 ≥ 6	X	XI	XII

Variation (v) was calculated by maximum score minus minimum score. Trend or tendency (diff) was determined by difference between two mean scores from 2-time frames (mean score of first frame – mean score of second frame). diff less than minus value (<-a) indicated increasing trend while diff equal to or more than plus value (a ≤) indicated decreasing trend. diff score between -a and a indicated stationary trend. Constant number a is different for each outcome. The constant number a was 1 for hospitalization and anti-TNF use while it was 3 for surgery. These constant numbers were selected to find the significant difference between groups. In those twelve patterns, increasing tendency/trend (I, IV, VII and X) or persistently fluctuating activity (XI) were classified as poor pattern whereas decreasing trend (III, VI, IX, and XII) or stable activity (II, V, and VII) were classified as good pattern.

v1, variation of first frame; v2, variation of second frame; diff, trend or tendency of activity.

Supplementary Table 2. Cox regression hazard analysis for the predictors of anti-TNF use

	Univariate analysis		Multivariate analysis	
Variables	Hazard ratio (95% CI)	p-value	Adjusted hazard ratio (95% CI)	p-value
Poor pattern (vs. good)	2.65 (1.09-6.45)	0.031	2.07 (0.81-5.24)	0.124
Female (vs. male)	1.83 (0.79-4.19)	0.153	1.64 (0.66-4.07)	0.277
Age at diagnosis <17 yr (vs. ≥17)	1.37 (0.51-3.69)	0.533	1.41 (0.49-4.01)	0.514
CDAI at enrolment (CDAI <150 vs. CDAI ≥150)	0.26 (0.10-0.63)	0.003	0.26 (0.10-0.65)	0.004
Behavior B1 (vs. B2 or B3)	0.35 (0.15-0.79)	0.012	0.33 (0.14-0.79)	0.013

CDAI, Crohn's disease activity index
